# On identity

**DOI:** 10.1186/1753-2000-7-24

**Published:** 2013-07-31

**Authors:** Klaus Schmeck, Jörg M Fegert, Susanne Schlüter-Müller

**Affiliations:** 1 Child and Adolescent Psychiatric Hospital, Psychiatric University Hospitals Basel, Basel, Switzerland; 2 Department Child and Adolescent Psychiatry and Psychotherapy, University Hospital Ulm, Ulm, Germany; 3 Practice for Child and Adolescent Psychiatry, Frankfurt, Germany; 4 University of Applied Sciences FHNW, Basel, Switzerland

## 

DSM-5 is on the market and found an enormous amount of attention in the media but also critique from scientists and clinicians. In the focus of the debate are reservations against establishing new disorders and lowering the threshold to diagnose mental disorders with the danger of mislabeling normal people [1]. The ambitious goal of the DSM-5 Task Force was to produce a shift of paradigm in psychiatry by introducing a dimensional approach. This was especially true for Axis II of DSM-IV-TR, the section of personality disorders that had been under debate for insufficient reliability and validity and for being too non-specific. This critique is not surprising bearing in mind that the general criteria for personality disorders in DSM-IV didn’t have a sufficient empirical basis. However, after long lasting controversies, the American Psychiatric Association (APA) Board of Trustees decided in December 2012 that DSM-5 maintains the categorical model and the DSM-IV-TR criteria of personality disorders. The alternative hybrid dimensional-categorical model is now included in a separate chapter in Section III of DSM-5 to encourage further empirical research [2]. Thus, a process of more than a decade’s intensive work has been stopped abruptly as the Personality Disorder Work Group was obviously not able to reach consensus or to convince the Board of Trustees of the superiority of the new model.

What are the main aims of a classification system? In clinical practice a classification system should guide clinicians to use the appropriate treatment approaches developed for specific disorders, and in research to yield a nosological system that helps to disentangle the complex etiology of mental disorders. For research in the field of personality disorders the decision to keep the old model with all its well-known shortcomings is a major step back, and it will amplify the shift away from the study of diagnoses towards a focus on dimensions of observable behavior and neurobiological measures as it is proposed by the NIMH in the Research Domain Criteria Project (RDoC) [3]. However, with the decision to keep the old system and to move the alternative model of personality disorders to another section of the manual clinicians and researchers will have the choice to use one or the other model. It will be of high interest to see which model will be preferred in the years to come.

Summing up the results of current empirical research the Personality Disorder Work Group has concluded that impairments in the self and interpersonal domains are most characteristic of personality disorders. Thus the “old-fashioned” construct of identity has been moved back on the stage. With a stable identity we experience ourselves as unique and we are sure of the boundaries between our self and that of others. Our ability to regulate a broad range of emotional experience aids to establish a stable self-esteem and to be accurate in our self-appraisal. Thus identity provides predictability and continuity of functioning and enables effective social exchanges.

The formation of a stable identity is one of the major developmental tasks that adolescents have to master on their way to become a mature adult. Most of us remember the more or less difficult years during this stage of development where we were no longer children but still not adults. Challenges like changing of the maturing body during puberty, first experiences of physical intimacy with romantic partners or academic and occupational choices can lead to more or less severe identity crises that often are part of this developmental stage. It is a common myth to assume that, as a consequence of this “adolescent turmoil”, personality is quite unstable during the years of adolescence and that a mature and stable personality starts around 18. A meta-analysis of the stability of personality traits during the life-span [4] yields quite a different picture of the story. In fact, trait consistency levels off between childhood (3–6 years: .52) and the college years (18–22 years: .51) with slightly lower stability scores during late childhood and adolescence.

If substantial changes in basic personality traits during adolescence are more fiction than fact, then we have to ask if impairment of personality development is also more stable than it has been assumed in former times. With respect to identity development we have to distinguish between temporary identity crises (that many clinicians call “adolescent crises”) and more stable identity impairment (“diffusion”). The need to reliably assess impairments of identity development lead to the construction of a new questionnaire, the AIDA (Assessment of Identity Development in Adolescents), that has been published in CAPMH in 2012 [5]. When several international working groups presented their results with this assessment tool at the IACAPAP conference in Paris in 2012 the idea for this special edition on “Identity” was born.

The first article approaches the subject from a philosophical point of view. Daniel Sollberger outlines the different philosophical meanings of the term identity in which the development of psychological identity concepts in the second half of the 20th century is embedded. He ends with some reflections on the role of identity in borderline personality disorder and the use of AIDA.

In his paper on “brain and self” Georg North off brings together expertise in psychiatry, philosophy and neuroscience. Modern brain imaging techniques yield fascinating possibilities to investigate the neural mechanisms underlying our subjective experience of a self. The results of neuroscientific studies on self and self-reference are compared to philosophical accounts, and their relevance for psychoanalytic approaches to self and ego are pointed out.

Klaus Schmeck and co-worker pick up the current debate on DSM-5 and the changes in the understanding of personality disorders that have led to the alternative model in section III of DSM-5, and demonstrate the relevance of impairments in identity for the understanding of personality disorders in adolescents.

In the next paper Jung and co-worker describe the results of the Basle research group on the assessment of identity development that follow-up the work presented in CAPMH in 2012 [5]. They demonstrate that the self-rating questionnaire AIDA has an excellent ability to distinguish adolescent patients with a DSM / ICD personality disorder diagnosis from both patients with internalizing or externalizing disorders and from non-referred youths so that the questionnaire can be used as screening tool in research on early starting personality disorders.

Since its development in 2011 the questionnaire AIDA has been translated and adapted for the use in many different countries. Besides the countries of origin (Switzerland, Germany and USA) these are (in alphabetical order): Bosnia-Herzegovina, Brazil, Chile, Croatia, Denmark, France, Greece, Kosovo, Lithuania, Mexico, Serbia and Spain. All these versions have been checked for psychometric properties or are currently under investigation. Culturally adapted versions are nearly ready in Hungary, Luxembourg, Netherlands, Singapore, Tunisia and Turkey. The president of IACAPAP, Prof. Omigbodun from Nigeria, currently supports the adaptation of AIDA for Nigeria and South-Africa.

The thorough review of the use of the Spanish version in different Spanish speaking countries (Chile, Mexico, Spain) revealed that slightly different formulations for some items are necessary to reach adequate reliability of the instrument. As an example of this multinational research Kassin and co-worker present reliability and validity data of the Mexican version that are studied in a school sample and a juvenile justice sample. Internal consistency reveals to be very good, and the questionnaire distinguishes adolescents from the different samples.

In spring 2013 CAPMH has become the official journal of the International Association for Child and Adolescent Psychiatry and Allied Professions (IACAPAP). Since the founding of CAPMH in 2007 the editors stimulated child psychiatrists and psychologists from all over the word to submit papers and they supported the preparation of these papers to become ready for publication. This article series is the first one conceptualized at a IACAPAP meeting. We are happy that this cooperation between IACAPAP and the journal carries its first scientific fruits.

## References

[B1] Frances A Saving Normal. An Insider’s Look at What caused the Epidemic of Mental Illness and how to cure It. William Morrow 2013

[B2] American Psychiatric Association Diagnostic and Statistical Manual of Mental Disorders 2013 5 Arlington VA: American Psychiatric Association

[B3] Insel T Research Domain Criteria (RDoC): Toward a New Classification Framework for Research on Mental Disorders Am J Psychiatry 2010 167 7 148 151 10.1176/appi.ajp.2010.09091379 20595427

[B4] Roberts BW DelVecchio WF The rank-order consistency of personality traits from childhood to old age: A quantitative review of longitudinal studies Psychol Bull 2000 126 1 3 25 10668348 10.1037/0033-2909.126.1.3

[B5] Goth K Foelsch PA Schlüter-Müller S Birkholzer M Jung E Pick O Schmeck K Assessment of identity development and identity diffusion in adolescence – Theoretical basis and psychometric properties of the self-report questionnaire AIDA Child and Adol Psych and Ment Heal 2012 6 27 http://www.capmh.com/content/6/1/27 10.1186/1753-2000-6-27 PMC3485126 22812911

